# Lineage- and developmental stage-specific mechanomodulation of induced pluripotent stem cell differentiation

**DOI:** 10.1186/s13287-017-0667-2

**Published:** 2017-09-29

**Authors:** Maricela Maldonado, Rebeccah J. Luu, Gerardo Ico, Alex Ospina, Danielle Myung, Hung Ping Shih, Jin Nam

**Affiliations:** 1 Department of Bioengineering, University of California-Riverside, Materials Science & Engineering Building 331, 900 University Avenue, Riverside, CA 92521 USA; 2 Department of Translational Research and Cellular Therapeutics, City of Hope, Duarte, California 91010 USA

**Keywords:** Induced pluripotent stem cells, Mechanobiology, Differentiation, Substrate stiffness

## Abstract

**Background:**

To maximize the translational utility of human induced pluripotent stem cells (iPSCs), the ability to precisely modulate the differentiation of iPSCs to target phenotypes is critical. Although the effects of the physical cell niche on stem cell differentiation are well documented, current approaches to direct step-wise differentiation of iPSCs have been typically limited to the optimization of soluble factors. In this regard, we investigated how temporally varied substrate stiffness affects the step-wise differentiation of iPSCs towards various lineages/phenotypes.

**Methods:**

Electrospun nanofibrous substrates with different reduced Young’s modulus were utilized to subject cells to different mechanical environments during the differentiation process towards representative phenotypes from each of three germ layer derivatives including motor neuron, pancreatic endoderm, and chondrocyte. Phenotype-specific markers of each lineage/stage were utilized to determine differentiation efficiency by reverse-transcription polymerase chain reaction (RT-PCR) and immunofluorescence imaging for gene and protein expression analysis, respectively.

**Results:**

The results presented in this proof-of-concept study are the first to systematically demonstrate the significant role of the temporally varied mechanical microenvironment on the differentiation of stem cells. Our results demonstrate that the process of differentiation from pluripotent cells to functional end-phenotypes is mechanoresponsive in a lineage- and differentiation stage-specific manner.

**Conclusions:**

Lineage/developmental stage-dependent optimization of electrospun substrate stiffness provides a unique opportunity to enhance differentiation efficiency of iPSCs for their facilitated therapeutic applications.

**Electronic supplementary material:**

The online version of this article (doi:10.1186/s13287-017-0667-2) contains supplementary material, which is available to authorized users.

## Background

The derivation of human induced pluripotent stem cells (iPSCs) has revolutionized the field of personalized regenerative medicine by offering a potentially unlimited cell source to treat a variety of diseases [1]. In addition to their clinical potential, human iPSCs provide opportunities to develop patient-tailored in vitro models for pathogenesis and toxicity studies [2–4]. To fully realize these diverse potentials, it is important to efficiently control iPSC behavior, such as self-renewal and differentiation [5]. Specifically, the ability to precisely modulate the lineage/phenotype-specific differentiation of cells is critical for minimizing safety concerns of iPSCs in vivo (e.g., tumorigenesis and teratoma formation) [6, 7] or generating physiologically relevant tissue models in vitro [2]. Similar to embryonic stem cells, iPSCs can be directed to differentiate towards various lineages/cell phenotypes by activating specific signaling pathways [7–9]. In particular, the sequential application of biochemical factors derived from embryonic development provided a foundational backbone to guide iPSC differentiation [10]. However, such protocols typically discount the role of the underlying physical factors, such as morphology, surface chemistry, and mechanical properties of substrates, which also affect differentiation efficiency.

In this regard, our previous studies have demonstrated the significant role of substrate stiffness on the spontaneous or directed early-stage differentiation of iPSCs [11, 12]. Electrospun nanofibers were used as a platform to control the stiffness of cell-adherent surfaces without affecting other physical parameters (e.g., surface chemistry, topography, availability of adhesion sites) to elicit mechanomodulated behaviors of iPSCs. The differences in substrate stiffness exerted profound effects on cell colony formation, resulting in various degrees of cell-material and cell-cell interactions, and ultimately the differentiation efficiency towards both mesendodermal and ectodermal lineages. In another study, we also showed the effects of substrate stiffness on the morphology of adult stem cells and their subsequent differentiation to end-phenotypes [13]. Based on those results, in this study we investigated the previously unexplored role of temporal changes in substrate stiffness on iPSCs throughout the course of differentiation. A well-characterized iPSC line [11] was differentiated towards representative phenotypes from each of the three germ layer derivatives while their substrate stiffness varied at each differentiation stage. Our results demonstrate that a sequential application of mechanically distinct electrospun substrates during the differentiation processes can enhance the differentiation efficiency of iPSCs in a lineage- and developmental stage-specific manner. This novel finding provides insights into the understanding of the mechanoresponsiveness of cell development and it establishes a platform to enhance directed differentiation of iPSCs by temporal modulation of the mechanical microenvironment.

## Materials and methods

### Electrospun substrate synthesis and characterization

Electrospinning conditions were optimized for 8 wt.% poly(ε-caprolactone) (PCL; Sigma-Aldrich, MO) dissolved in 5:1 trifluoroethanol-water and 5 wt.% polyether-ketone-ketone (PEKK; Oxford Performance Materials, CT) dissolved in 1,1,1,3,3,3-hexafluoro-2-propanol (HFP; Oakwood Products Inc., SC) to synthesize nanofibrous substrates with approximately 450-nm fiber diameter. To obtain the same surface chemistry for cell adhesion, the substrates were air plasma-treated and collagen-conjugated as previously described [12] (see Additional file 1 for chemical characterization of the scaffolds). The elemental composition of the electrospun substrate surface was characterized by x-ray photoelectron spectroscopy (XPS) using a Kratos AXIS ULTRADLD XPS system equipped with an Al Kα monochromated x-ray source and a 165-mm mean radius electron energy hemispherical analyzer. The substrates were also mechanically characterized using atomic force microscopy (AFM) as previously described [12]. The surface area was measured by the Brunauer-Emmett-Teller (BET) method using nitrogen adsorption.

### Cell culture and differentiation

A well-characterized human iPSC line, derived from BJ-2522 human neonatal foreskin fibroblast cells transfected with OCT4, SOX2, and KLF4 as previously described [11], was maintained on Geltrex®-coated tissue culture polystyrene plates (TCPS) in mTeSR™1 medium (Stemcell Technologies, Canada) in a humidified incubator at 37 °C and 5% CO_2_. Cells were passaged from tissue culture plates for each stage of differentiation using 0.25% Trypsin-EDTA (Life Technologies, NY). A ROCK inhibitor, Y-27632 (EMD Millipore, MA), was used at 10 μM concentration to enhance cell survival after seeding onto electrospun substrates or TCPS. After overnight incubation, the Y-27632 was removed and fresh media supplemented with lineage- and stage-specific growth factors was added. Media was exchanged daily unless otherwise noted. To examine the effects of temporally varied electrospun substrate stiffness on the differentiation of iPSCs, step-wise differentiation protocols for motor neuron, pancreatic endoderm, and chondrocytes were utilized. Details of the differentiation protocols and analysis of gene and protein expression at each differentiation stage can be found in the supporting information (see Additional file 2).

## Results and discussion

**Fig. 1:**
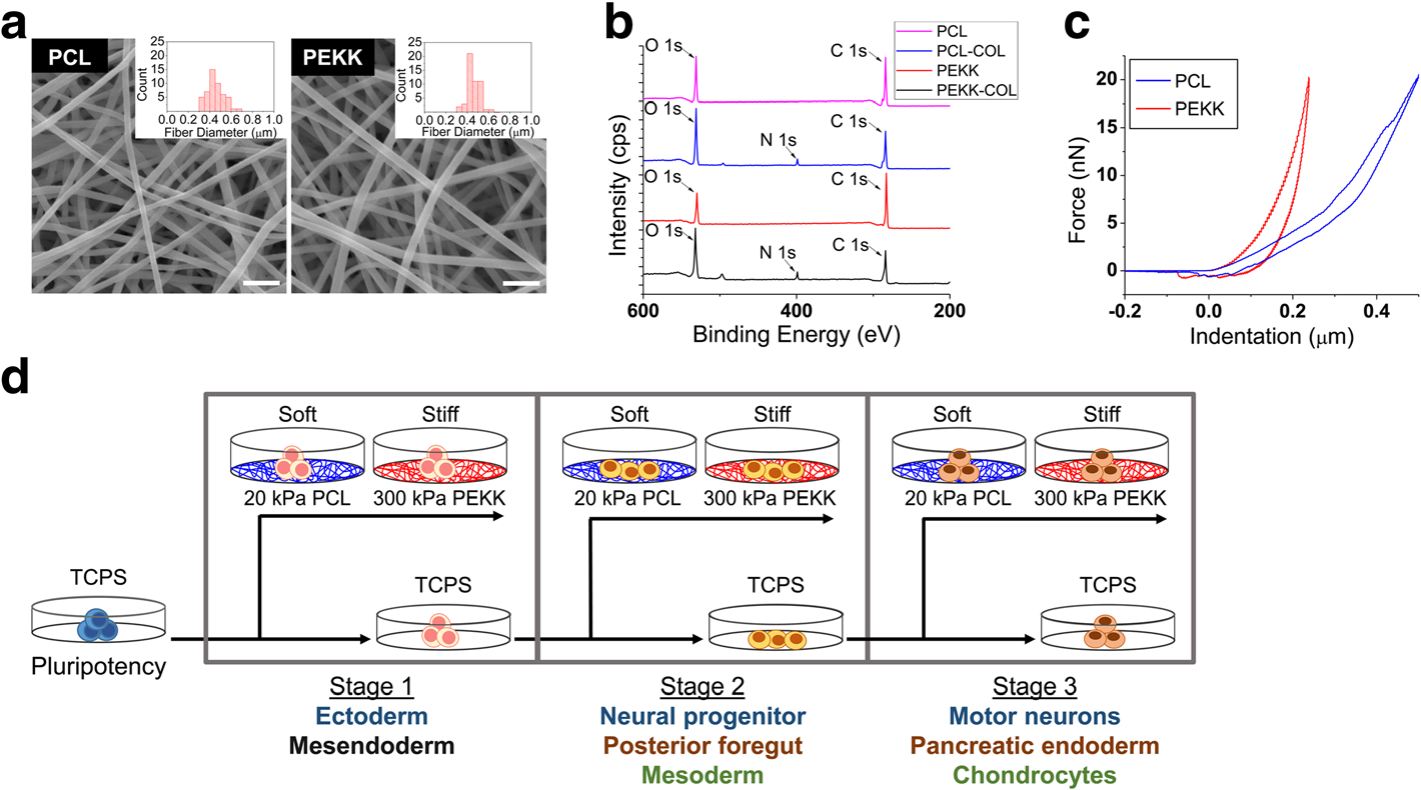
Characterization of electrospun substrates and experimental schematic to examine the effects of temporally varied substrate stiffness. **a** Scanning electron micrographs and fiber diameter histograms (*insets*) of electrospun poly(ε-caprolactone) (*PCL*) and polyether-ketone-ketone (*PEKK*) substrates (*scale bar* = 2 μm). **b** XPS spectra of electrospun PCL and PEKK substrates with/without collagen (*COL*) conjugation. **c** Representative force-indentation curves of electrospun PCL and PEKK substrates by atomic force microscopy. **d** A schematic of the experimental design to examine the effects of temporally varied substrate stiffness on each developmental stage of differentiation. Human iPSCs were differentiated along three phenotypes from each germ layer lineage via sequential supplementation of biochemicals (e.g., Stage 1: ectoderm; Stage 2: neural progenitor; Stage 3: motor neurons). Human iPSCs were cultured on tissue culture polystyrene plates (*TCPS*) prior to passaging for Stage 1 differentiation to either mesendoderm or ectoderm on soft (PCL) or stiff (PEKK) electrospun substrates. Alternatively, cells were continuously cultured on TCPS during Stage 1 differentiation before being seeded onto the soft or stiff electrospun substrates for further differentiation to Stage 2. Similar passaging and differentiation on either TCPS or the soft/stiff substrates were performed for Stage 3. At the end of each differentiation stage, the samples were analyzed for gene and protein expression of lineage/developmental stage-specific markers

Electrospun scaffolds provide a means to examine the contributions of substrate stiffness on stem cell differentiation while uniquely regulating cell/colony morphologies [12, 13]. To synthesize mechanically distinctive, yet morphologically and surface chemically similar substrates, we optimized the electrospinning process of PCL or PEKK to achieve average fiber diameters of approximately 486 ± 128 nm for PCL and 476 ± 77 nm for PEKK (Fig. 1a). The surface area of PCL and PEKK scaffolds was determined to be 2.70 m^2^/g and 2.85 m^2^/g, respectively, by the BET method. Taking into account the similar density of PCL (1.145 g/cm^3^) and PEKK (1.278 g/cm^3^) and the similar fiber diameters, we estimate a similar porosity between the scaffolds. Collagen type I was chemically conjugated to these substrates via zero-length crosslinking by NHS/EDAC, and their surface modification was confirmed by XPS and immunofluorescent microscopy (Fig. 1b). The XPS spectra of collagen-conjugated electrospun PCL and PEKK nanofibers show the presence of nitrogen (N_1s_) at 400 eV, specific to the amino acids of collagen, in addition to the elemental peaks of the polymers of O_1s_ and C_1s_ at the binding energies of 530 and 284 eV, respectively. The 10-μm thick substrates were mechanically characterized by atomic force microscopy to calculate the reduced Young’s moduli of approximately 20 kPa for PCL and 300 kPa for PEKK from force-indentation curves (Fig. 1c). To examine the effects of temporally varied substrate stiffness on the differentiation efficiency of iPSCs at different developmental stages, the cells were cultured on either the soft (20 kPa) or stiff (300 kPa) substrates during differentiation towards motor neuron, pancreatic endoderm, or chondrocyte identities (Fig. 1d).

Cells cultured on different substrates (i.e., soft PCL, stiff PEKK or TCPS control) at each differentiation stage were subjected to defined growth factors [14–16]. Briefly, motor neuron differentiation was induced by initial dual inhibition of SMAD signaling [17] followed by patterning to motor neurons using brain-derived neurotrophic factor (BDNF), ascorbic acid, sonic hedgehog (SHH), and retinoic acid [14]. Pancreatic endoderm differentiation was induced by high concentrations of activin A followed by primitive gut tube, posterior foregut, and pancreatic endoderm/endocrine precursor induction using fetal bovine serum (FBS), KAAD-cyclopamine, and retinoic acid [15]. Finally, chondrocyte differentiation was initiated by primitive streak induction with Wnt3a/activin A, followed by mesoderm induction using bone morphogenetic protein 4 (BMP4) and basic fibroblast growth factor (FGF2), and chondrogenesis by decreasing BMP4 and increasing growth differentiation factor 5 (GDF5) concentrations [16]. Each of the biochemically driven protocols was optimized to yield the highest population of cells expressing lineage/developmental stage-specific markers to ultimately enhance the overall differentiation efficiency. However, such step-wise protocols typically do not examine how different substrate stiffness affects differentiation at each stage, and forego the opportunity to further enhance the differentiation efficiency via optimization of the physical cell niche.

**Fig. 2:**
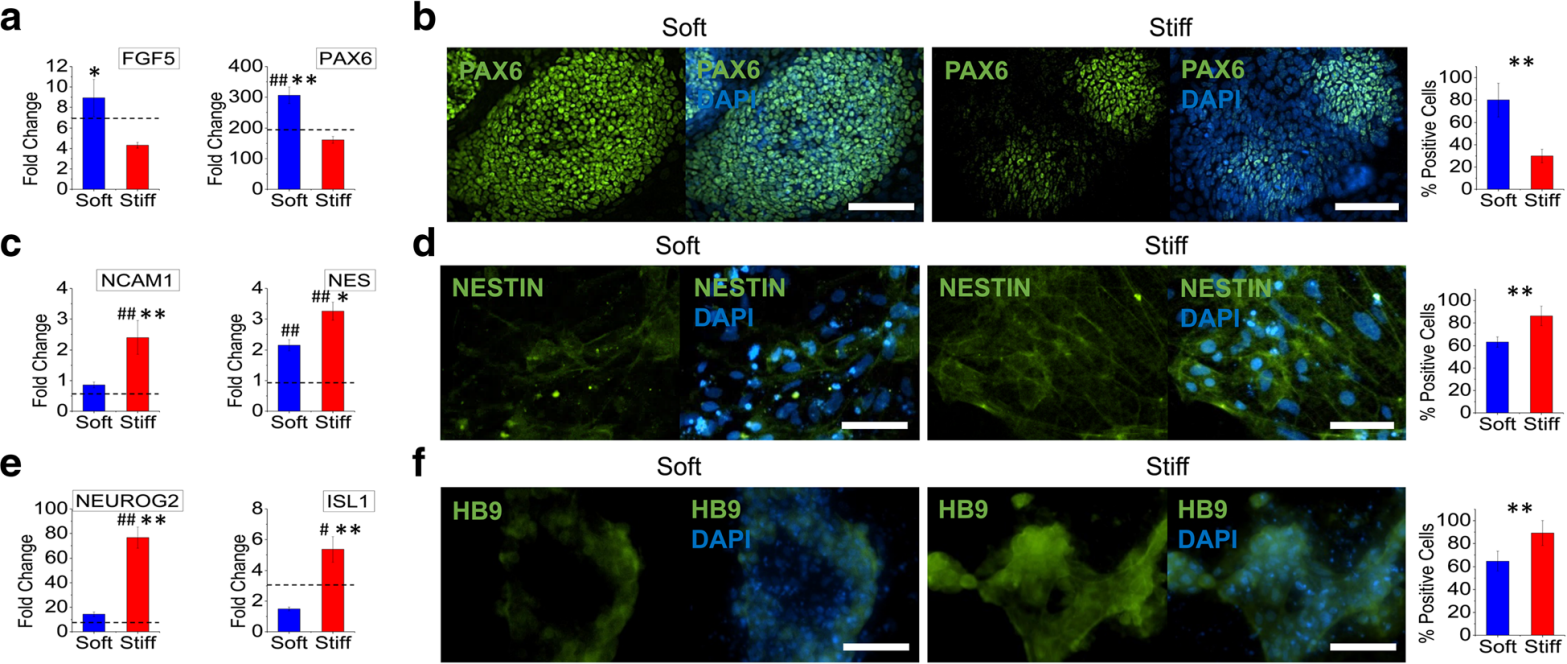
The stage-specific effects of substrate stiffness on motor neuron differentiation. Human iPSCs were differentiated on either soft (PCL) or stiff (PEKK) electrospun substrates to (**a**, **b**) ectodermal, (**c**, **d**) neural progenitor, or (**e**, **f**) motor neuron lineage. **a** Gene expression of ectodermal markers *FGF5* and *PAX6* was significantly upregulated on soft substrates as compared to stiff substrates. **b** Immunofluorescent imaging and quantification of percent-positive cells showed that PAX6 protein expression was significantly higher on soft substrates after ectodermal induction (*green*: PAX6; *blue*: DAPI; *scale bar* = 100 μm). **c** Gene expression of neural progenitor markers *NCAM1* and *NES* was significantly upregulated on stiff substrates as compared to soft substrates. **d** Immunofluorescent imaging and quantification of percent-positive cells showed that NESTIN protein expression was higher on stiff substrates (*green*: NESTIN; *blue*: DAPI; *scale bar* = 100 μm). **e** Gene expression of motor neuron markers *NEUROG2* and *ISL1* was significantly upregulated on stiff substrates as compared to soft substrates. **f** Immunofluorescent imaging and quantification of percent-positive cells showed that HB9 protein expression was higher on stiff substrates (*green*: HB9; *blue*: DAPI; *scale bar* = 100 μm). Gene expression was normalized to that of cells from the preceding stage of differentiation cultured on TCPS (fold change = 1). The *dashed line* represents the average fold change of differentiated cells on TCPS. ^#^ *p* < 0.05, ^##^ *p* < 0.01, versus TCPS differentiated controls. *p < 0.05, **p < 0.01, between substrates

We first examined how electrospun substrate stiffness affected the differentiation of iPSCs to motor neurons (Fig. 2). During the first stage of differentiation towards ectodermal lineage from the pluripotent state, the expression of ectodermal markers *FGF5* and *PAX6* was significantly increased on the soft substrates (Fig. 2a). PAX6, at the protein level, also showed enhanced expression on the soft substrates with a 50% increase in percent-positive PAX6 cells as compared to those on the stiff substrates (Fig. 2b). To further examine the effects of substrate stiffness on the downstream differentiation, ectodermal cells were subcultured onto either soft or stiff substrates for subsequent neural progenitor differentiation. Unlike the previous differentiation stage, the differentiation efficiency of ectodermal cells to neural progenitors was enhanced on the stiff substrate (Fig. 2c and d). A significant increase in gene expression of *NCAM1* and *NES* (Fig. 2c) and protein expression of NESTIN (Fig. 2d) was observed when cells were cultured on the stiff substrates. The final downstream specification of neural progenitors towards motor neurons was similarly enhanced on the stiff substrates as evident from significant increases of motor neuron markers *NEUROG2* and *ISL1* at the gene level and HB9 at the protein level on the stiff substrates (Fig. 2e and f). Unlike studies using hydrogel systems where neurogenesis is enhanced on softer substrates, our results indicate that specification of neural progenitor cells to motor neurons is enhanced on stiffer substrates [18, 19]. Inherent differences in topography and the pliability of electrospun fiber networks, in addition to the tested stiffness range and the specified differentiation stage, may collectively contribute to this discrepancy. Nevertheless, the results presented here demonstrate the mechanoresponsive nature of iPSCs at the early stages of lineage commitment where ectodermal induction is enhanced on soft substrates while the downstream specification to neural progenitors or motor neurons is enhanced on stiffer electrospun substrates.

**Fig. 3:**
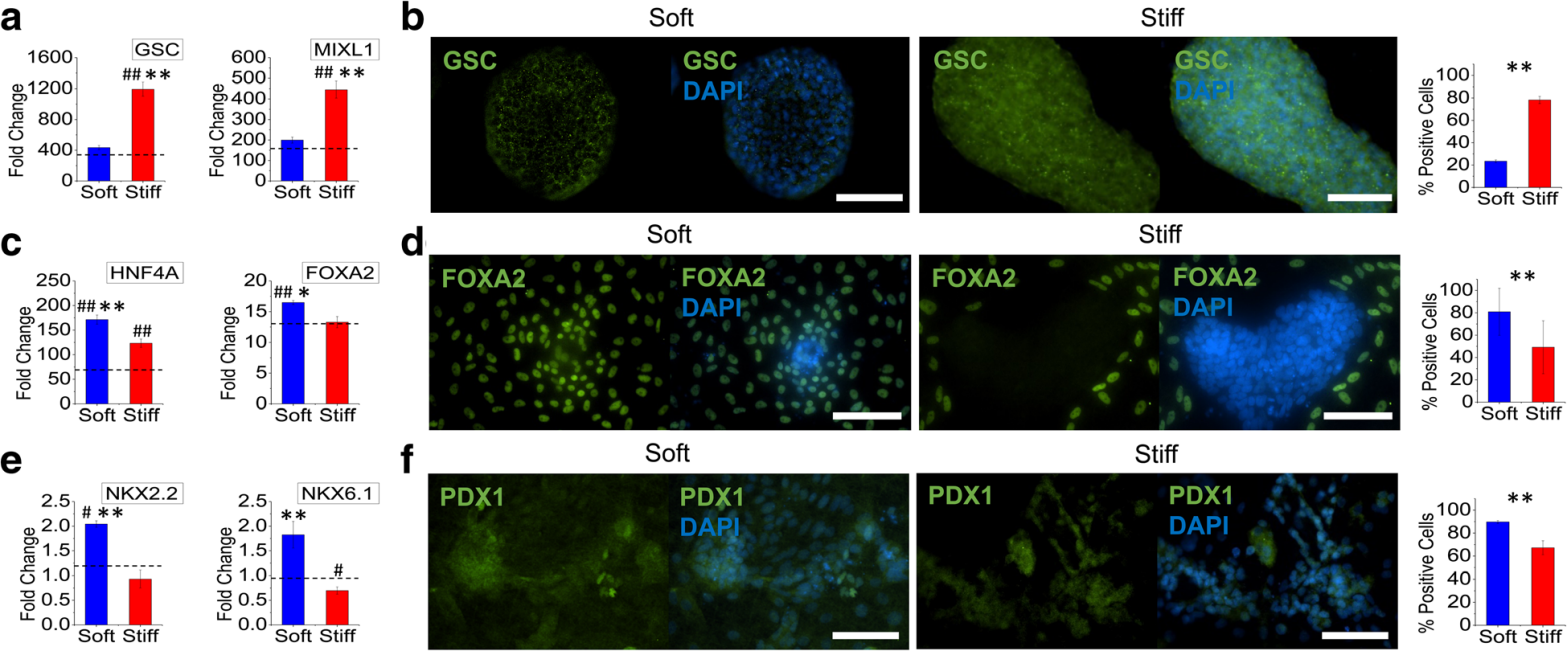
The stage-specific effects of substrate stiffness on pancreatic endoderm differentiation. Human iPSCs were differentiated on either soft (PCL) or stiff (PEKK) electrospun substrates to (**a**, **b**) mesendodermal, (**c**, **d**) posterior foregut, or (**e**, **f**) pancreatic endoderm lineage. **a** Gene expression of mesendodermal markers *GSC* and *MIXL1* was significantly upregulated on stiff substrates as compared to soft substrates. **b** Immunofluorescent imaging and quantification of percent-positive cells showed that GSC protein expression was significantly higher on stiff substrates (*green*: GSC; *blue*: DAPI; *scale bar* = 100 μm). **c** Gene expression of posterior foregut markers *HNF4A* and *FOXA2* was significantly upregulated on soft substrates as compared to stiff substrates. **d** Immunofluorescent imaging and quantification of percent-positive cells showed that FOXA2 protein expression was significantly higher for cells cultured on soft substrates (*green*: FOXA2; *blue*: DAPI; *scale bar* = 100 μm). **e** Gene expression of pancreatic endoderm markers *NKX2.2* and *NKX6.1* was significantly upregulated on soft substrates as compared to stiff substrates. **f** Immunofluorescent imaging and quantification of percent-positive cells showed that PDX1 protein expression was higher on soft substrates (*green*: PDX1; *blue*: DAPI; *scale bar* =100 μm). The *dashed line* represents the average fold change of differentiated cells on TCPS. ^#^ *p* < 0.05, ^##^ *p* < 0.01, versus TCPS differentiated controls. *p < 0.05, **p < 0.01, between substrates

To examine the endodermal lineage differentiation of iPSCs, we alternatively directed the cells towards pancreatic endoderm specification. In contrast to early ectodermal differentiation, expression of mesendodermal markers *GSC* and *MIXL1* was significantly increased on the stiff substrates as compared to both the soft substrates and TCPS controls when iPSCs were subjected to differentiation towards mesendodermal lineage (Fig. 3a). GSC expression at the protein level was also significantly enhanced on the stiff substrates (Fig. 3b). On the other hand, the subsequent differentiation of mesendodermal cells to posterior foregut was significantly enhanced on the soft substrates evident by the greater gene expression of *HNF4A* and *FOXA2* (Fig. 3c). In accordance, protein expression of FOXA2 was significantly enhanced on the soft substrates showing 32% greater percent-positive cells as compared to those on the stiff substrates (Fig. 3d). The final specification of posterior foregut cells to pancreatic endoderm was also significantly enhanced on the soft substrates evident from the gene expression of pancreatic markers *NKX2.2* and *NKX6.1* and the protein expression of PDX1 (Fig. 3e and f).

**Fig. 4:**
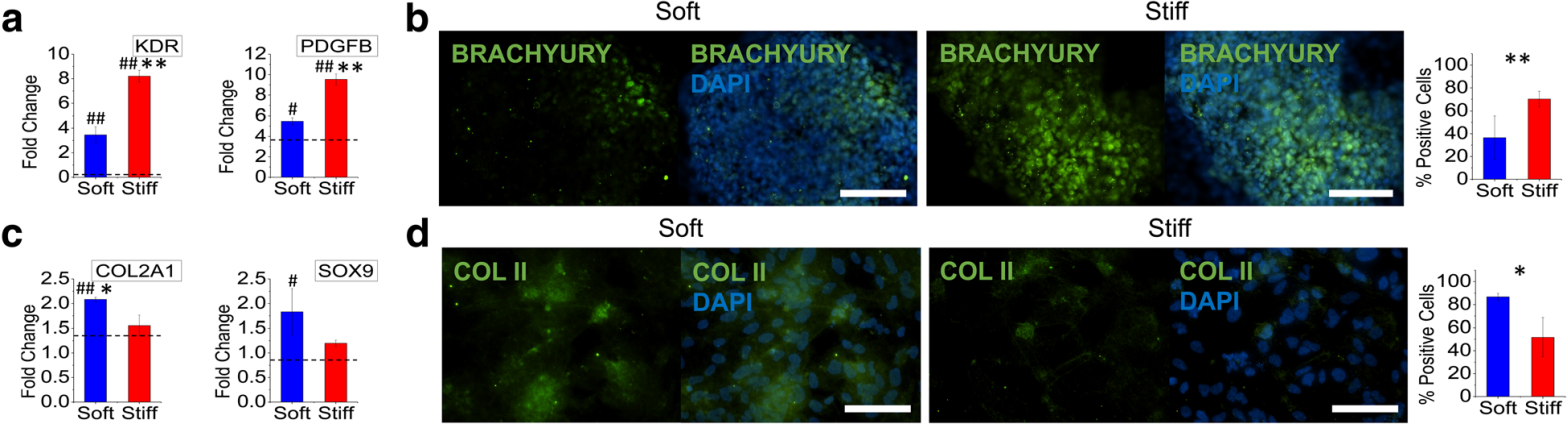
The stage-specific effects of substrate stiffness on chondrocyte differentiation. Human iPSCs were differentiated on either soft (PCL) or stiff (PEKK) electrospun substrates to (**a**, **b**) mesoderm or (**c**, **d**) chondrocyte lineage. **a** Gene expression of mesoderm markers *KDR* and *PDGFB* was significantly upregulated on stiff substrates as compared to soft substrates. **b** Immunofluorescent imaging and quantification of percent-positive cells showed that BRACHYURY protein expression was significantly higher on stiff substrates (*green*: BRACHYURY; *blue*: DAPI; *scale bar* = 100 μm). **c** Gene expression of chondrocyte markers *COL2A1* and *SOX9* was upregulated on soft substrates as compared to stiff substrates. **d** Immunofluorescent imaging and quantification of percent-positive cells showed that collagen type II protein expression was significantly higher on soft substrates (*green*: COL II; *blue*: DAPI; *scale bar* = 100 μm). The *dashed line* represents the average fold change of differentiated cells on TCPS. ^#^ *p* < 0.05, ^##^ *p* < 0.01, versus TCPS differentiated controls. *p < 0.05, **p < 0.01, between substrates

In spite of the same origin with posterior foregut, the differentiation of mesendodermal cells to mesodermal specification was preferred on the stiff substrates (Fig. 4a and b). Gene expression of mesodermal markers *KDR* and *PDGFB* was significantly enhanced on the stiff substrates as compared to both the soft substrates and TCPS control (Fig. 4a). Additionally, protein expression of BRACHYURY was also enhanced with a significant increase of 33% positive cells on the stiff substrates as compared to the soft substrates (Fig. 4b). These results suggest that the mechanical microenvironment contributes to the divergence of a mesendoderm population where specification of endodermal phenotypes is enhanced on soft substrates whereas mesodermal phenotypes are enhanced on stiff substrates. Similarly, the specification of mesodermal cells to chondrocytes was significantly enhanced on the soft substrates (Fig. 4c and d). Gene expression of *COL2A1* and *SOX9* was significantly increased on the soft substrates, with protein expression of collagen type II also displaying a similar trend (Fig. 4c and d). Taken together, these results suggest that the final stage of downstream differentiation to either pancreatic endoderm or chondrocyte shares the same substrate stiffness-dependent differentiation behaviors although a divergence in optimal substrate stiffness was observed for the preceding differentiation stage. Chondrocyte commitment from a mesodermal stage is consistent with our previous work with mesenchymal stem cells where chondrogenesis was enhanced on a soft electrospun substrate [13].

## Conclusions

Overall, the results presented in this proof-of-concept study are the first to systematically demonstrate the significant role of the temporally varied mechanical microenvironment on the differentiation of stem cells in a lineage- and developmental stage-specific manner. For example, neural induction is initially enhanced on soft substrates but, as the differentiation process progresses, stiff substrates promote neural progenitor and motor neuron specification. In contrast, mesendodermal differentiation is significantly enhanced on stiff substrates but further specification to posterior foregut requires a soft substrate, indicating that dynamic changes of substrate stiffness may further enhance differentiation efficiency. Although optimization of the differentiation protocol was beyond the scope of this study, the optimal combination of the substrate stiffness examined in this study (e.g., sequential application of soft-stiff-stiff substrates for each stage of ectodermal-neural progenitor-motor neuron differentiation) is estimated to achieve an approximately 5-, 8-, and 11-fold increase in the yield of differentiated cells in neural, pancreatic endoderm, and chondrocytic phenotypes, respectively. Collectively, these results suggest that optimization of the mechanical microenvironment using electrospun nanofibrous substrates, incorporated into biochemically driven differentiation protocols, provides an efficient method of producing therapeutic cells for translational applications of iPSCs.

## Additional files

Additional file 1: Chemical characterization of electrospun scaffolds. Fluorescence images of nanofibrous scaffolds that are collagen type I-conjugated or unconjugated and stained with an anticollagen type I antibody. (PDF 186 kb)

Additional file 2: Supporting information on Materials and methods. Detailed materials and methods for the differentiation of iPSCs towards various lineages, gene/protein expression analysis, statistical analysis, and a table of RT-PCR primers. (PDF 395 kb)

## Abbreviations

AFM: Atomic force microscopy
BDNF: Brain-derived neurotrophic factor
BET: Brunauer-Emmett-Teller
BMP4: Bone morphogenetic protein 4
FBS: Fetal bovine serum
FGF2: Fibroblast growth factor
GDF5: Growth differentiation factor 5
HFP: 1,1,1,3,3,3-hexafluoro-2-propanol
iPSC: Induced pluripotent stem cell
PCL: Poly(ε-caprolactone)
PEKK: Polyether-ketone-ketone
SHH: Sonic hedgehog
TCPS: Tissue culture polystyrene plates
XPS: X-ray photoelectron spectroscopy
