# Supplementary material for: Lineage- and developmental stage-specific mechanomodulation of induced pluripotent stem cell differentiation
Source: Stem Cell Res Ther. 2017 Sep 29;8:216. doi: 10.1186/s13287-017-0667-2 (PMC5622562; doi:10.1186/s13287-017-0667-2)
Supplement: Supplementary file 1 — Chemical characterization of electrospun scaffolds. Fluorescence images of nanofibrous scaffolds that are collagen type I-conjugated or unconjugated and stained with an anticollagen type I antibody. (PDF 186 kb) [file 13287_2017_667_MOESM1_ESM.pdf]

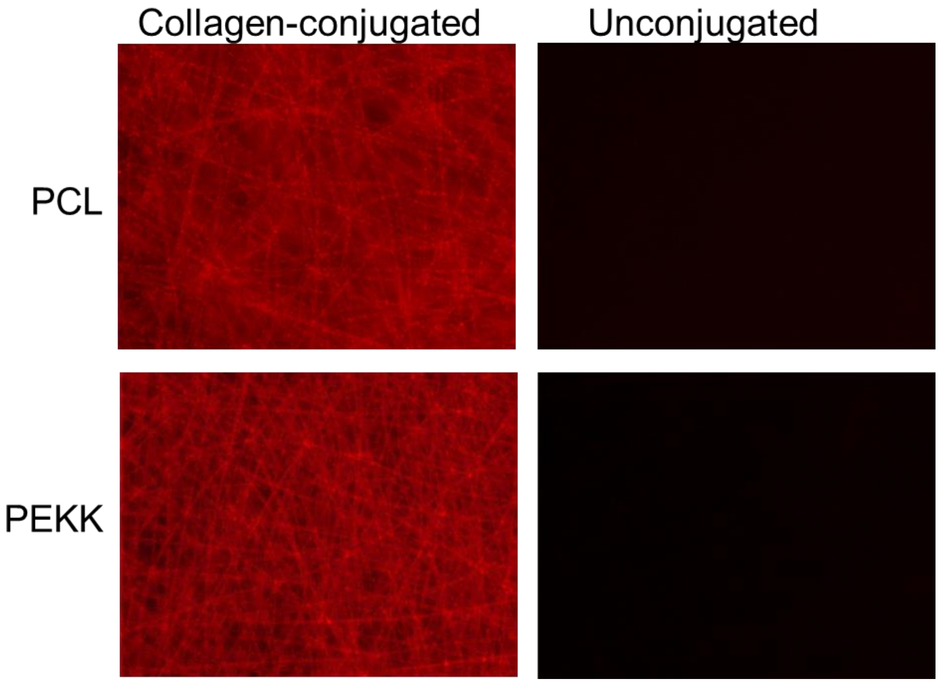

Chemical characterization of electrospun scaffolds.

Nanofibrous scaffolds were collagen type I-conjugated or unconjugated and stained with an anti-collagen type I antibody.
