# Supplementary material for: Lineage- and developmental stage-specific mechanomodulation of induced pluripotent stem cell differentiation
Source: Stem Cell Res Ther. 2017 Sep 29;8:216. doi: 10.1186/s13287-017-0667-2 (PMC5622562; doi:10.1186/s13287-017-0667-2)
Supplement: Supplementary file 2 — Supporting information on Materials and methods. Detailed materials and methods for the differentiation of iPSCs towards various lineages, gene/protein expression analysis, statistical analysis, and a table of RT-PCR primers. (PDF 395 kb) [file 13287_2017_667_MOESM2_ESM.pdf]

Supporting Information

Lineage- and developmental stage-specific mechano-modulation of induced pluripotent stem cell differentiation

Maricela Maldonado[1], *Rebeccah J. Luu*[1], *Gerardo Ico*[1], *Alex Ospina*[1], *Danielle Myung*[1], *Hung-Ping Shih*[2] *and Jin Nam*[1*]

[1]Department of Bioengineering, University of California, Riverside, California 92521

[2]Department of Translational Research and Cellular Therapeutics, City of Hope, Duarte, California 91010

* Corresponding author: Jin Nam, PhD

jnam@engr.ucr.edu

Department of Bioengineering

University of California-Riverside

Materials Science & Engineering Building 331

900 University Avenue

Riverside, CA, 92521

TEL: 951-827-2064

FAX: 951-827-6416

MATERIALS AND METHODS

Differentiation of iPSCs

To study the effects of electrospun substrate stiffness on the differentiation of iPSCs, defined developmental-stage protocols for motor neuron, pancreatic endoderm, and chondrocytes were utilized. The iPSCs were pre-cultured on Geltrex®-coated tissue culture plates (TCPS) according to the protocol recommendations (i.e., motor neuron differentiation required a three day proliferation culture period to reach near confluency prior to the induction of differentiation). Initially, iPSCs were seeded at 70,000 cells/cm^2 on TCPS for propagation and induction of differentiation to stage 1, 2, or 3. Alternatively, to analyze the effects of substrate stiffness at stage 1 of differentiation, iPSCs were seeded directly onto electrospun substrates. The iPSCs which were differentiated on TCPS through stage 1 were seeded onto electrospun substrates for differentiation through stage 2. Similarly, cells differentiated on TCPS through stage 2 were seeded onto substrates for differentiation through stage 3. At the end of each differentiation stage, samples were either lysed for gene expression analysis or fixed for protein expression analysis.

Differentiation to motor neurons

To differentiate iPSCs towards a motor neuron lineage, a protocol by Chambers et al. was utilized to guide the cells through the ectodermal, neural progenitor, and motor neuron stages [1]. Media was supplemented every other day with stage-specific media and growth factors. For stage 1 differentiation the cells were cultured in knockout replacement (KO) media (Gibco) supplemented with 10 µM TGF-β inhibitor, SB431542 (Cayman Chemical, MI) and 500 ng/ml Noggin (PeproTech, NJ) (days 1-5). Stage 2 differentiation was induced on days 6-10 by gradually increasing the amount of N2 media to 25%, 50%, and 75%. The media was

supplemented with 20 ng/ml BDNF (Peprotech), 0.2 mM ascorbic acid (Sigma), 200 ng/ml sonic hedgehog (Peprotech), and 0.1 µM retinoic acid (Sigma). Stage 3 differentiation was induced on days 11-19 using N2 media supplemented with 20 ng/ml BDNF, 0.2 mM ascorbic acid, 200 ng/ml sonic hedgehog, and 0.1 µM retinoic acid.

Differentiation to pancreatic endoderm

A protocol by Kroon et al. was utilized to differentiate iPSCs through mesendoderm, posterior foregut, and pancreatic endoderm stages [2]. Media was supplemented daily with stage-specific media and growth factors. For stage 1 differentiation the cells were cultured in RPMI Glutamax media (Gibco) supplemented with 100 ng/ml Activin A (PeproTech) and 25 ng/ml Wnt3a on day 1 and RPMI media with 0.2% vol/vol FBS supplemented with 100 ng/mL Activin A on days 2-4. At the start of stage 2, the wells were briefly washed with PBS containing calcium and magnesium and media was exchanged to RPMI media with 2% vol/vol FBS supplemented with 25-50 ng/ml KGF on days 5-9. On days 10-12 the media was changed to DMEM with 1% vol/vol B27 supplemented with 0.25 µM KAAD-cyclopamine (EMD Millipore, CA), 2 µM retinoic acid, and 50 ng/ml noggin. Stage 3 was induced on days 13-16 by DMEM with 1% vol/vol B27 and no additional growth factors.

Differentiation to chondrocytes

The directed differentiation of iPSCs to chondrocytes was adapted from a protocol by Oldershaw et al. by guiding the cells through a mesendoderm, mesoderm, and chondrocyte stage [3]. Basal media consisting of DMEM:F12 with L-glutamine, ITS, nonessential amino acids, B27, and beta-mercaptoethanol, was exchanged daily with stage-specific growth factors. Stage 1 was induced by the addition of: 25 ng/ml WNT3A and 50 ng/ml Activin A on day 1; 25 ng/ml WNT3A, 25 ng/ml Activin A, and 20 ng/ml FGF2 on day 2; 25ng/ml WNT3A, 10 ng/ml Activin

A, 20 ng/ml FGF2, and 40 ng/ml BMP4 on day 3. Stage 2 was induced by the addition of: 20 ng/ml FGF2, 40 ng/ml BMP4, 100 ng/ml Follistatin, and 2 ng/ml NT4 on days 4-7; 20 ng/ml FGF2, 40 ng/ml BMP4, and 2 ng/ml NT4 on day 8. Stage 3 was induced by the addition of: 20 ng/ml FGF2, 20 ng/ml BMP4, 20 ng/ml GDF5, and 2 ng/ml NT4 on days 9-10; 20 ng/ml FGF2, 40 ng/ml GDF5, and 2 ng/ml NT4 on days 11-14.

Gene and protein expression analysis

The cells were harvested at the end of each differentiation stage for total RNA extraction using an RNeasy Micro Kit (Qiagen, CA) following manufacturer instructions. Complementary DNA (cDNA) synthesis was performed using an iScript cDNA Synthesis Kit (Bio-Rad, CA). Quantitative real-time PCR was utilized to detect stage/lineage specific markers using custom primers (**Table S1**). Gene expression levels were analyzed using the comparative threshold cycle (CT) method with *GAPDH* used as an endogenous control[4].

To analyze the protein expression of stage/lineage-specific markers, cells were fixed with 4% paraformaldehyde for 30 minutes followed by staining using standard protocols. The respective primary antibodies: mouse anti-PAX6 (Developmental Hybridoma Studies Bank (DSHB), IA), mouse anti-GSC (Abcam, CA), mouse anti-Nestin (DSHB), goat anti-FOXA2 (R&D systems, MN), goat anti-BRACHYURY (R&D Systems), mouse anti-HB9 (DSHB), rabbit anti-PDX1 (Abcam), or mouse anti-Collagen Type II (DSHB) and secondary antibodies: donkey anti-mouse 488, donkey anti-goat 488, or goat anti-rabbit 488 (Invitrogen, MA) were used for immunocytochemical staining. Samples were counter-stained using mounting medium with 4',6-diamidino-2-phenylindole (DAPI) (Vectashield, Vector Laboratories, CA) to stain nuclei. Image J software was used to quantify the marker protein-positive cells using the single color images to count total cell number (DAPI images) and the protein-positive cells (green fluorescence

images), as previously described [5]. Briefly, the images were converted to an 8-bit image type and the threshold was adjusted to highlight the cell nuclei. To divide merged cells the image was processed using the Watershed option. The 'analyze particles' function was used to determine the cell count. If the clarity of the images taken was not sufficient to accurately obtain a cell count the nuclei or positive cells were counted manually. At least 10 images were analyzed from stage-specific stained samples and the data is presented as mean ± standard deviation.

Statistical analysis

All experiments were conducted with at least triplicate samples and data is represented as means ± standard error of means (SEM) unless otherwise noted. Statistical significance was determined by the one sample student T-test using SPSS (v.23.0) software. A '*p*' value of 0.05 or less was considered statistically significant.

TABLE S1. Custom real-time polymerase chain reaction (PCR) primers.

Sequences of forward and reverse primers for lineage-specific genes.

Primer	Forward	Reverse
GAPDH	*5′*-GCAAATTCCATGGCACCGT-*3′*	*5′*-TCGCCCCACTTGATTTTGG-*3′*
	Ectoderm	
FGF5	*5′*-GTATGTGGCCCTGAATAAAAGAGG-*3′*	*5′*-AAAGTTCTGGCTGCTCCGACT-*3’*
PAX6	*5′*-GAGTTCTTCGCAACCTGGCTA-*3′*	*5′*-CTGCCCGTTCAACATCCTTAG-*3’*
NES	*5′*- CACCTGTGCCAGCCTTTCTTA-*3′*	*5′*- TGGAGCAGAGAGAGAGGAGCAT-*3’*
VIM	*5′*-GGAAGAGAACTTTGCCGTTGAAG-*3′*	*5′*-ACGAAGGTGACGAGCCATTTC-*3’*
NEUROG2	*5′*- CATCAAGAAGACCCGTAGACTGA-*3′*	*5′*-TCTCGATCTTGGTGAGCTTGG-*3’*
ISL1	*5′*-GCTTACAGGCTAACCCAGTGGAA-*3′*	*5′*-TGTCACTCTGCAAGGCGAAGTC-*3′*
	Mesendoderm	
GSC	*5′*-GATGCTGCCCTACATGAACGT-*3′*	*5′*-TACTTGGTCTCCTGGAAGAGGTT-*3′*
MIXL1	*5′*-CTTTGGCTAGGCCGGAGATTA-*3′*	*5′*-GGCAGGCAGTTCACATCTACCT-*3′*
	Endoderm	
HNF4A	*5′*- TCCAAAACCCTCGTCGACAT-*3′*	*5′*- TTGCCCATCGTCAACACCT-*3′*
FOXA2	*5′*- TCCATCAACAACCTCATGTCCT-*3′*	*5′*- CATCACCTGTTCGTAGGCCTTG-*3′*
NKX2.2	*5′*-ACGCAGGTCAAGATCTGGTTC-*3′*	*5′*-GCGTCACCTCCATACCTTTCTC-*3′*
NKX6.1	*5′*-CCACTTTTTCCGGACAGCA-*3′*	*5′*-CCCCAACGAATAGGCCAAA-*3′*
	Mesoderm	
KDR	*5′*-CACCACTCAAACGCTGACATGTA-*3′*	*5′*-CCAACTGCCAATACCAGTGGA-*3′*
PDGFB	*5′*-ATGCAGACATCGAGTCCTCCA-*3′*	*5′*-ATCAAAGTTGCTCGGCAGGTC-*3′*
COL2A1	*5′*-CAACACTGCCAACGTCCAGAT-*3′*	*5′*-CTGCTTCGTCCAGATAGGCAA-*3′*
SOX9	*5′*-TGCTCAAAGGCTACGACTGGA-*3′*	*5′*-TTGACGTGCGGCTTGTTCT-*3′*

REFERENCES

1. Chambers, S.M., et al., Highly efficient neural conversion of human ES and iPS cells by dual inhibition of SMAD signaling. Nat Biotechnol, 2009. **27**(3): p. 275-80.
2. Kroon, E., et al., Pancreatic endoderm derived from human embryonic stem cells generates glucose-responsive insulin-secreting cells in vivo. Nat Biotechnol, 2008. **26**(4): p. 443-52.
3. Oldershaw, R.A., et al., Directed differentiation of human embryonic stem cells toward chondrocytes. Nat Biotechnol, 2010. **28**(11): p. 1187-94.
4. Livak, K.J. and T.D. Schmittgen, Analysis of relative gene expression data using real-time quantitative PCR and the 2(-Delta Delta C(T)) Method. Methods, 2001. **25**(4): p. 402-8.
5. Nam, J., et al., Modulation of embryonic mesenchymal progenitor cell differentiation via control over pure mechanical modulus in electrospun nanofibers. Acta Biomater, 2011. **7**(4): p. 1516-24.
